# Large apparent growth increases in boreal forests inferred from tree-rings are an artefact of sampling biases

**DOI:** 10.1038/s41598-019-43243-1

**Published:** 2019-05-02

**Authors:** Louis Duchesne, Daniel Houle, Rock Ouimet, Liam Caldwell, Manuel Gloor, Roel Brienen

**Affiliations:** 1 Ministère des Forêts, de la Faune et des Parcs, Direction de la recherche forestière, 2700 Einstein Street, Quebec City, Quebec G1P 3W8 Canada; 2 Consortium on Regional Climatology and Adaptation to Climate Change (Ouranos), 550 Sherbrooke Street West, Montreal, Quebec H3A 1B9 Canada; 3 School of Geography, University of Leeds, Leeds, LS2 9JT UK

**Keywords:** Plant ecology, Forest ecology, Population dynamics

## Abstract

Tree rings are thought to be a powerful tool to reconstruct historical growth changes and have been widely used to assess tree responses to global warming. Demographic inferences suggest, however, that typical sampling procedures induce spurious trends in growth reconstructions. Here we use the world’s largest single tree-ring dataset (283,536 trees from 136,621 sites) from Quebec, Canada, to assess to what extent growth reconstructions based on these - and thus any similar - data might be affected by this problem. Indeed, straightforward growth rate reconstructions based on these data suggest a six-fold increase in radial growth of black spruce (*Picea mariana*) from ~0.5 mm yr^−1^ in 1800 to ~2.5 mm yr^−1^ in 1990. While the strong correlation (R^2^ = 0.98) between this increase and that of atmospheric CO_2_ could suggest a causal relationship, we here unambiguously demonstrate that this growth trend is an artefact of sampling biases caused by the absence of old, fast-growing trees (cf. “*slow-grower survivorship bias*”) and of young, slow-growing trees (cf. “*big-tree selection bias*”) in the dataset. At the moment, we cannot envision how to remedy the issue of incomplete representation of cohorts in existing large-scale tree-ring datasets. Thus, innovation will be needed before such datasets can be used for growth rate reconstructions.

## Introduction

Anthropogenic greenhouse gas emissions have resulted in an unprecedented rise in atmospheric CO_2_ over the past decades^1–3^ and have led to rapid global warming^4^. Understanding the response of forests to these changes is important, as forests play a crucial role in the global carbon cycle^5–7^. However, evidence for long-term changes in growth, and possible drivers and mechanisms such as CO_2_ fertilization and temperature increases, remains poor. Changes occurring at northern latitudes may be of particular importance, as these regions experience the fastest rates of warming and are the second largest above-ground organic carbon store, after tropical forests^8^. Reported recent growth trends in this region disagree emphasizing the need for a better understanding of long-term historical changes in land vegetation cover and its functioning. This is especially important to predict the role played by forests in moderating future atmospheric greenhouse gas levels.

All approaches used to study the response of forests to environmental changes have their strengths and limitations. Among all, tree-ring data have the advantage of extending far back in time. In addition, they provide naturally annual records and can easily be obtained. They have thus been seen as a prime candidate to provide a long-term perspective of tree growth response to global environmental change^9,10^. Hence, such data have been used more and more over recent years for growth reconstructions^11–21^.

However, there is a large, underestimated problem with tree-ring data, namely that tree-ring sampling procedures can lead to spurious growth trends as sampled trees are not representative of the full cohorts of trees that lived in the past^22–25^. This is particularly apparent in tree-ring studies in which only living, dominant and codominant trees are sampled^22,23,25^. Among the various potential biases (see^24,26^), those arising from differences in tree growth rates have received much attention. In particular, if longevity differs between fast- and slow-growing trees, then biases may arise. Indeed, evidence suggests that fast-growing trees, which reach their maximum potential size sooner than slow-growing trees have a shorter lifespan^27–29^. Therefore, sampling only currently living trees leads to an underrepresentation of fast-growing trees in the earlier portion of the growth dataset, as those early born, fast- growing trees would have died before sampling (cf. “*slow-grower survivorship bias”*)^23^. As a result, tree-ring based growth reconstructions extending over long periods tend to be biased toward slow growth in the early part of the record. This creates an apparent increase in growth rate over time. In addition, classic tree-ring sampling approaches disproportionately favor larger trees, which introduces another bias. Young, slow-growing trees will not have reached the sampling diameter threshold, whereas fast-growing trees of the same age are larger and thus more likely to be sampled (cf. “*big-tree selection bias”*)^23^. As a result, fast-growing trees will be overrepresented in the more recent period. This further accentuates the apparent trend of increased tree growth in the most recent period. The existence of these biases has been demonstrated theoretically, using stochastic tree growth simulations^23^, and in real nature, for one single, small stand-scale tree-ring dataset^25^. However, we know little about how common these biases are, more generally, and to what degree they affect growth trend reconstructions in large-scale studies.

**Figure 1:**
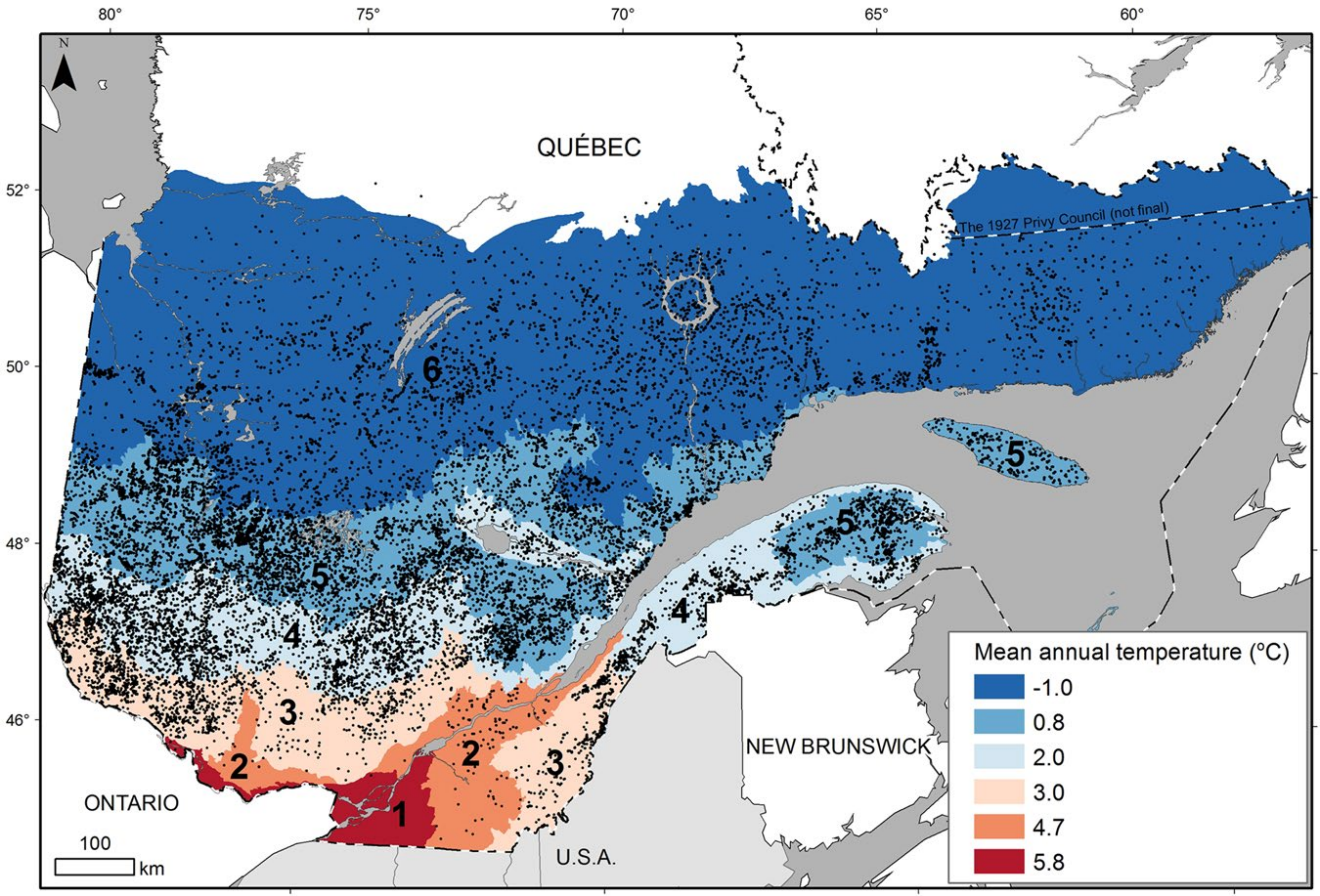
Location of sample plots (black dots n = 33,711) in Quebec (Canada) where black spruce trees were sampled for tree-ring measurements. The numbers correspond to bioclimatic domains which were mainly associated with variations in mean annual temperature along the latitudinal gradient of the studied territory. 1: sugar maple-bitternut hickory domain; 2: sugar maple-basswood domain; 3: sugar maple-yellow birch domain; 4: balsam fir-yellow birch domain; 5: balsam fir-white birch domain; 6: spruce-moss domain. Map created using ArcGIS (version10.3) software from Esri inc. (http://www.esri.com/arcgis/). Data for base maps from https://www.donneesquebec.ca/recherche/fr/dataset/systeme-hierarchique-de-classification-ecologique-du-territoire and https://www.donneesquebec.ca/recherche/fr/dataset/base-de-donnees-geographiques-et-administratives-a-lechelle-de-1-1-000-000 used with permission under a Creative Commons 4.0—Attribution CC BY.

In this study, we use a unique dataset of tree-ring measurements from 283,536 trees sampled in 136,621 stands to analyse trends in radial growth over the last 250 years and assess to what extent growth reconstructions based on these data might be affected by the above-mentioned biases. This dataset comprises the world’s largest number of tree-ring samples collected according to standardised methods across a large geographic region (eastern Canada). We focus our analysis first on black spruce (*Picea mariana*), which is the most abundant tree species in this dataset (Fig. 1), and then verify if the same pattern can be observed for 12 other tree species.

We here also test to what degree biases arise when using different tree-ring detrending approaches. The study of external (climatic) forcing on tree growth requires the removal of age- or size- related variation in growth rates, commonly referred to as ‘tree-ring standardisation’^30^. Various methods have been used to remove age -or size-related growth trends^31^. Traditional standardisation methods fit a curve to individual series and remove all variance at the individual tree level, including a substantial part of the long-term historical growth rate variation, which is exactly the variation of interest for studies on the tree’s response to CO_2_ and global warming. Thus, a growing number of studies have developed and used more conservative standardisation methods that maintain growth difference between trees (e.g. regional curve standardisation)^32,33^ or focused on raw, unstandardised or basal-area-converted ring widths time series^15–17,20,21,34–39^. Given that the magnitude of biases may differ depending on the choice of detrending method, we compare three different detrending methods. First, we simply focus on a fixed diameter or age range (cf. size or age class isolation detrending)^31^ to avoid the effect of declining radial growth with increasing tree age and size^32,33^. Then, we repeat this analysis using two common tree-ring standardisations, a traditional tree-level standardisation procedure (c.f. linear regression standardisation)^30^ and the increasingly advocated Reginal Curve Standardisation (RCS)^33^.

## Results and Discussion

### Growth trends

**Figure 2:**
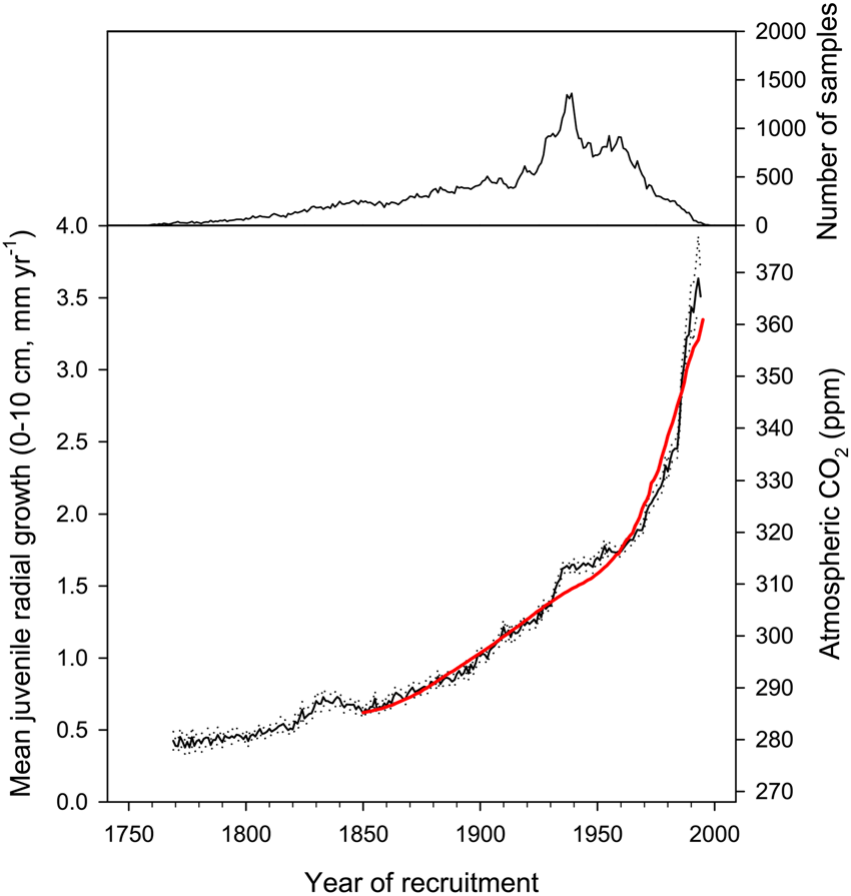
Time series of average mean annual radial growth of trees at the juvenile stage (diameter: 0–10 cm) from yearly successive generations of black spruce trees (lower graph, black line), along with change in atmospheric CO_2_ concentrations (lower graph, red line). Dotted lines delimit 95% confidence intervals. The growth series was truncated when sample replication dropped below 25. The upper graph illustrates the number of samples in each generation. Estimated values for atmospheric CO_2_ concentrations were taken from^3^. The growth increase is deceptively similar to the increase in atmospheric CO_2_ (R^2^ = 0.98).

Simple averaging of juvenile growth rate of black spruce cohorts over the last 250 years indicates a six-fold increase in ring widths from means (±95% CI) of 0.40 ± 0.03 mm for tree cohorts in the 1760s to 2.63 ± 0.03 mm for those in the 1980s (Fig. 2). This rapid nonlinear growth increase is deceptively similar to the increase in atmospheric CO_2_ since pre-industrial times (R^2^ = 0.98, Fig. 2). Thus, it is tempting to attribute this trend to the effect of global change on forest productivity (through temperature changes or CO_2_ fertilization, for example). However, further analysis reveals that this interpretation is erroneous.

### Sample biases

**Figure 3:**
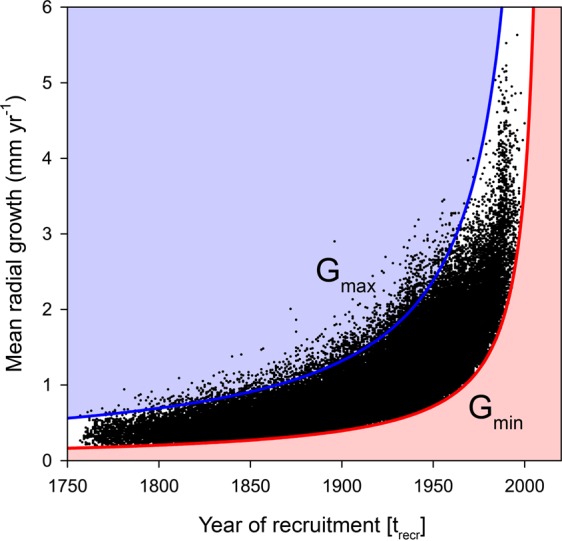
Lifetime mean annual radial growth of individual black spruce trees (n = 85,982) aligned by year of recruitment (pith year). The upper blue line corresponds to the maximum radial growth rates as a function of recruitment years, G_max_ (t_recr_) = D_max_/(2012 − t_recr_); the lower red line corresponds to the minimum growth rate at which a tree can reach the minimum diameter threshold, G_min_ (t_recr_) = D_thresh_/(2012 − t_recr_). Trees growing faster than G_max_ (t_recr_) (blue line) would have reached diameters larger than the 99 percentile size (D_max_), and are unlikely to be still alive in 2012. The absence of fast growers from the distant past (blue shaded area) has been previously described as the “*slow grower survivorship bias*”^23^. The red shaded area corresponds to missing slow-growing trees that grew at rates slower than G_min_ (t_recr_) and did not reach the minimum size threshold (D_thresh_) (i.e., “*big-tree selection bias”*)^23^.

Assuming that a species-specific, size-dependent mortality rate exists and that the vast majority of trees dies before reaching a given ‘maximum’ diameter, then there will be an inverse relationship between growth rate and longevity (i.e., fast-growing trees will have a shorter lifespan than slow-growing trees)^27–29^. To estimate to which degree the difference in life-expectancy of fast- and slow-growing trees may have driven our results, we assumed that there exists a maximum diameter, D_max_, which trees reach before they die. We then estimated G_max_, the limiting maximum growth rate allowing trees to be still alive in 2012, the last year of sampling, as a function of past recruitment time (t_recr_):

1 $${{\rm{G}}}_{{\rm{\max }}}\,({{\rm{t}}}_{{\rm{recr}}})={{\rm{D}}}_{{\rm{\max }}}/(2012-{{\rm{t}}}_{{\rm{recr}}}),$$

where t_recr_ is given in years AD. We estimated D_max_ as the 99^th^ percentile of diameters in the dataset, which is 298 mm diameter for black spruce (see Fig. S1). From the curve of G_max_ as a function of recruitment year (Fig. 3, blue line), it is clear that there is a strong asymmetry across past time in growth rates of trees that are expected to be still alive in 2012 (all trees with growth rates and recruitment ages in the area below the blue line). Thus, among trees recruited far in the past, only very slow-growing trees will be part of living trees at a given year of sampling.

Another bias arises because living trees are usually not sampled uniformly across diameter classes. Indeed, the sampling protocol used in the field (see methods) probes only trees that have reached 91 mm in diameter (or 45.5 mm in radius) at a height of 1.3 m above ground level, thus excluding those individuals which have not yet reached the minimum diameter threshold (D_thresh_). In an approach similar to the one above, we can express G_min_, the minimum mean growth rate which a tree needs to reach this minimum diameter threshold, as a function of recruitment time in the past:

2 $${{\rm{G}}}_{{\rm{\min }}}({{\rm{t}}}_{{\rm{recr}}})={{\rm{D}}}_{{\rm{thresh}}}/(2012-{{\rm{t}}}_{{\rm{recr}}}).$$

Again, there is a strong asymmetry among trees with different growth rates across recruitment times in the past that make it into the sample of trees probed. This is because only trees with growth rates and recruitment ages above the red line in Fig. 3 will be sampled. Indeed, when recruitment years and mean growth rate of all black spruce trees of our dataset are added in the same figure (Fig. 3) they mostly populate the area between the red and blue lines. This gives strong support for our interpretation of the effects of a growth longevity trade-off and of a minimum sampling size threshold on growth rate reconstructions. Since the increasing trend of the blue and red curves does not result from any growth stimulation, it appears very likely that the convex shapes of growth vs. age relationships in Figs 2 and 3 are simply an artefact induced by these biases. The exact same pattern was observed for 12 other common boreal tree species showing that the bias observed for black spruce is widespread (Fig. S2).

### Growth-longevity trade-off

**Figure 4:**
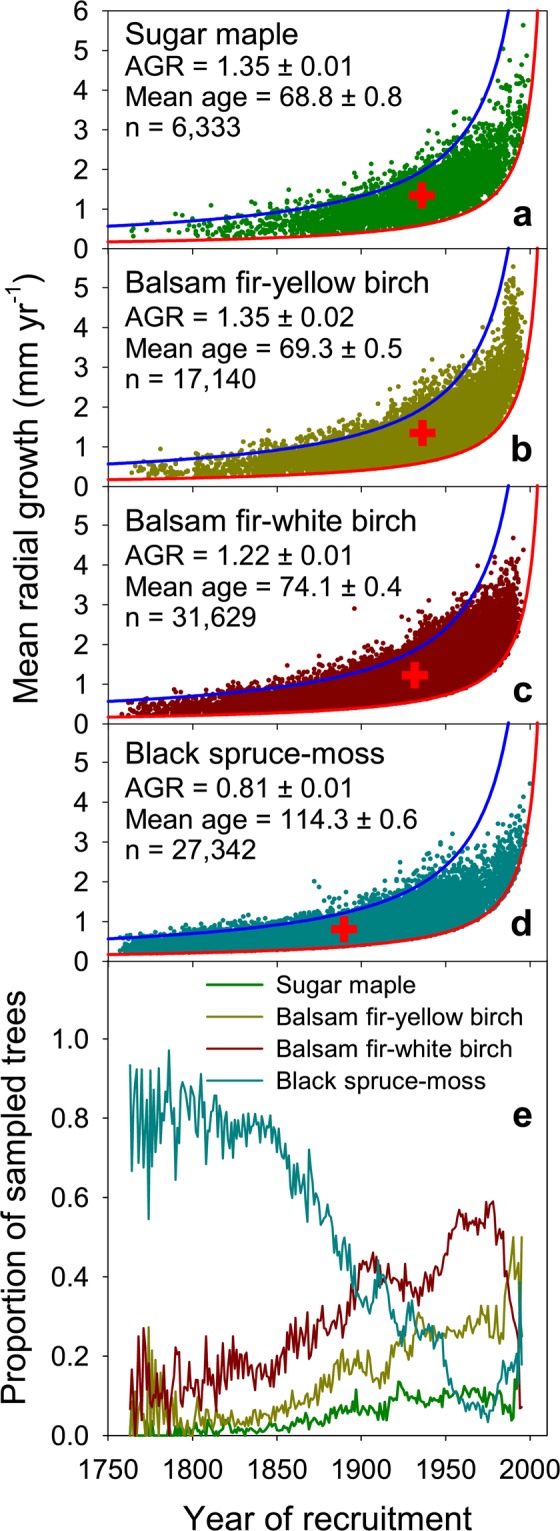
Lifetime mean annual radial growth of individual black spruce trees aligned by year of recruitment (pith year, panels a–d) and proportion of sampled trees from yearly generations according to their location (bioclimatic domains, panel e, see Fig. 1). Red crosses indicate the mean annual radial growth rates and mean year of recruitment for each subset. Mean annual growth rate (AGR, mm yr^−1^), mean tree age (yr) and their 95% confidence intervals, and sample size (n) are given for each subset. See the legend of Fig. 3 for definition of blue and red lines in panels (a–d). Older tree cohorts are biased toward a higher proportion of slow-growing trees originating from less productive sites, while younger generations are biased toward a higher proportion of fast-growing trees from more productive sites. These biases induce spurious trends in historical growth rate reconstruction. The proportion series were truncated when sample replication dropped below 25.

The black spruce tree-ring dataset covers more than 33,000 sites over a large geographic region. It is thus ideal to probe the extent to which growth rates and tree age covary with environmental growing conditions, and to evaluate how this may cause biases in growth rate reconstructions. If growth rates and tree age are inversely related across our dataset, we expect to observe temporal shifts in the relative proportions of trees belonging to zones with different productivity (growth rates) in our sample. Indeed, we find that in our dataset tree growth in the northern and colder bioclimatic domain are lower and that trees reach older ages (114 years) when compared with southern and warmer territories, where growth rates are higher and tree ages are lower (69 years) (Fig. 4a–d). As a result of this, we see shifts in the relative proportions of trees in our dataset coming from these different zones over time, with a higher proportion (~80%) of slow-growing trees from less productive colder sites in the earlier period (e.g. 1760–1850), and higher proportions of fast-growing trees from warmer sites in recent periods (Fig. 4e). Similar patterns emerge when trees are stratified according to soil or crown cover with decreases in the proportion of trees growing on less productive soils (very shallow or very stony soils and soils with hydric moisture regime) and of trees from less productive, open lichen–spruce woodlands (26–40% crown cover) (Fig. S3e,j). These results indicate either that there are true strong temporal shifts in the relative recruitment of black spruce in different subregions, or that trees on more productive sites live shorter lives. Given the consistency in shifts of relative abundance across subregions stratified using intrinsically different environmental characteristics (i.e., climate, soil, crown cover), we argue that these changes in relative proportion of trees in the dataset coming from different subregions are indeed due to a trade-off between growth and longevity. Shorter tree longevity at the most productive sites results in lower abundance of trees at these sites in earlier periods. While this must drive part of the observed convex relationship between growth and recruitment year, restricting the dataset to a single bioclimatic zone, soil physical environment, or stand density class still results in strong biases in growth reconstructions (See Fig. S4). Similarly, we find that the biases still persist even when separating the dataset by the plot levels of disturbances, such as fire, or intervention history (thinning or harvesting) (Fig. S5). This analysis provides yet another warning against the use of tree-ring data to infer patterns of phenomena such as historical recruitment across large geographic regions or over long time periods^40,41^, as tree lifespan depends on productivity, and thus varies between sites.

### Relative contribution of biases

**Figure 5:**
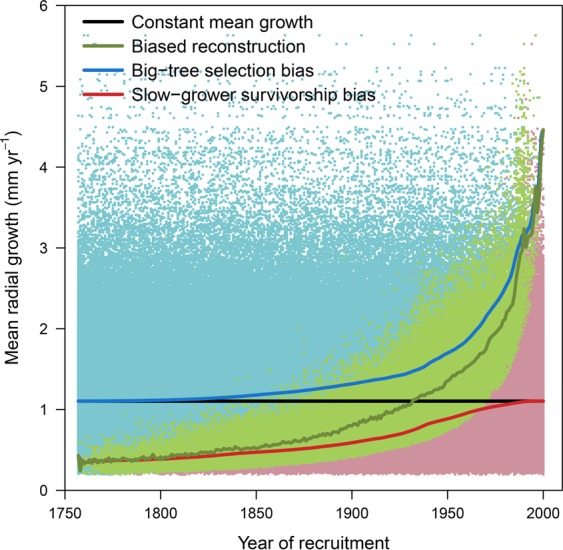
Schematic illustration of biases in reconstructions of black spruce growth. All trees estimated to have been once present in the population have been considered, including in green, trees sampled in this study (same as in Fig. 3) and in red and blue respectively, trees that were excluded because they were smaller than 91 mm at the time of sampling (cf. “*big-tree selection bias*”), and because they died before sampling (cf. “*slow-grower survivorship bias*). The black line shows the unbiased growth reconstruction of all trees historically present in the simulation (all dots), the red line shows the trend arising due to the slow-grower survivorship bias, and the blue line shows the bias due to the big-tree selection bias. This analysis shows that the spurious trends from Fig. 3 are for about 80% driven by the big-tree selection bias and for the remainder by the slow-grower survivorship bias. A subset of only 1 million randomly selected individual values are shown for clarity.

To disentangle the relative contribution of the two biases, caused by absence of old, fast-growing trees and of young, slow-growing trees from the dataset, to the growth trends, we created a hypothetical steady state tree growth trajectory dataset. This dataset was constructed by shifting backwards and forwards in time, the lifetime mean annual radial growth data of all individual black spruce samples one year at a time for the period of 1757 to 2000 (from Fig. 3). By construction, for such a tree population, growth rate is constant over time (horizontal black line in Fig. 5, growth rate 1.1 mm yr^−1^). However if we sample only trees living in the year 2000 and restrict ourselves to trees with diameters larger than 91 mm, we observe a rapidly increasing mean radial growth rate (green data points, Fig. 5). If we now include all trees living in 2000 with diameters smaller than 91 mm (red dots in Fig. 5), the very large bias (‘*big-tree selection bias*’) during the more recent period (last 100 years) disappears. On the other hand, if we include all trees which were historically present but have died before 2000 (but not the trees alive in 2000 with a diameter less than 91 mm) then the large bias (‘*slow-grower survivorship bias*’) at the far end of the period (1750–1800) disappears. In summary, this analysis shows that the spurious trend in our data (green line in Fig. 5) is approximately to 80% driven by the big-tree selection bias (blue line) with the remainder driven by the slow-grower survivorship bias (red line). This is consistent with our previous predictions using stochastic tree growth simulations showing that the big-tree selection bias has the largest effect^23^. Importantly, even when including small trees, and thus avoiding the big tree selection bias altogether, we still find a five-fold increase in growth rates since 1750 due to the slow-grower survivorship bias. Nehrbass-Ahles *et al*.^25^ recommend that sampling all individuals in a population would satisfy the requirements for all growth studies, including long-term growth trend reconstructions, but our results clearly contradict this assertion.

### Effect of data standardisation

 Tree ring based studies aiming to estimate tree growth response to global environmental changes commonly apply standardisation methods, which are essential for the interpretation of tree-ring data in the context of global change responses. However, as we will demonstrate they either do not remove the two biases we describe here, or do remove the biases at the cost of losing the long-term signal. Existing standardisation procedures aiming at removing biological effects on growth rates due to tree ageing, or size-related changes in tree physiology^31^, can be classified roughly into two categories: (i) “*population- or plot-level*” detrending approaches, such as “Regional Curve Standardisations”, RCS^32,33^ or size class isolation methods^31^, which use a population or site-specific size- or age-related growth curve to detrend the individual tree series, and (ii) “*tree-level*” detrending approaches which use linear or negative exponential functions, or cubic smoothing splines to detrend individual tree-ring series^34,42–45^. In both approaches, growth rate anomalies are calculated as the difference (or ratio) between the original tree-ring width time series and the fitted (detrending) curve. The main differences between these approaches are the frequency band of variation retained and their susceptibility to the biases we described.

We examined the interplay of the main standardisation methods and biases described here. Firstly, our approach to examine growth in a particular size class falls into the first category, “*population level detrending”*. We had focused on only the smallest size class, and extended this to two larger size classes and to three different age classes (Fig. S6). This analysis shows that biases arise in all scenarios, but to a different degree (Fig. S6). The magnitude of the bias in ring width decreases slightly for larger size classes due to a diminished effect of the big-tree selection bias (cf. ref. ^23^). Using age classes instead of size classes shows largely similar patterns as using size classes (Fig. S6b). The observed biases also persist and are in some cases even greater when expressing growth as basal area increment instead of ring width increment, showing that apparent growth trends also depends strongly on the choice of growth metrics used (Fig. S6). Using a similar and widely applied approach for *population level detrending*, the Regional Curve Standardisation^32,33^, shows also strong biases (see Fig. S7c). In contrast, the second approach, *tree level detrending,* does fully remove the bias (see Fig. S7d), but it also loses the ability to detect historical long-term growth changes that extend beyond the length of individual growth series. Various intermediate approaches have been applied recently, such as using generalized additive mixed models to predict individual growth series from regional or plot-level expectation of age trends (modeled by negative exponential smoothing, for example)^14,46^, or using individual or multiple RCS curves according to individual growth rates of trees, plots, or sites (*adaptive regional growth curve*)^47^. Some of these approaches do remove part or most of this bias, but only at the cost of losing information about long-term growth rate changes. Unfortunately, no single detrending procedure yet exists that is able to simultaneously maintain long-term variation and correct for the bias observed here.

### Challenge

Some procedures such as sampling all living trees in a fixed plot, reduces the big-tree selection bias, but our results show that such adjustments of field sampling designs (cf. ref.^25^) do not resolve the problem arising from the trade-off between growth and longevity. At the moment, we see no fix to the problem arising from missing growth information of trees that died during the period of the growth reconstruction. Only a complete sample of the entire historical population allows truly unbiased reconstructions. Theoretically, this problem could be solved by sampling trees that died recently and are still easily recognisable or sub-fossil trees, stumps and logs^23–25^. However finding and sampling all individuals that were once present in the population is nearly impossible, and we thus conclude that systematic biases in historical growth rate reconstruction at the population- or stand-level are practically unavoidable. We therefore recommend great caution in interpreting low-frequency variation associated with historical growth reconstructions from tree rings of the types presented here.

In addition to the two biases detected here, other types of biases due to a non-uniform age structure of sampled trees (“*non-uniform age bias*”)^26^ and possible declines in growth rates of trees prior to death (“*pre-death suppression bias*”)^24^ may induce apparent negative growth trends over time. This further complicates the use of tree-ring data for long-term growth and climate reconstructions. Hence, preserving long-time scale environmentally related changes in tree-ring width data while removing age- and size- dependent variations and disentangling positive and negative biases remains one of the biggest challenges for historical growth rate and climate reconstructions^22,23,48–50^. We challenge theorists and demographers to come up with solutions to these problems. In the meantime, our results raise doubts about the validity of existing studies that suggest growth increases based on tree-ring analyses.

## Methods

The study area corresponds to the forest territory below the current northern limit of the managed forest in Quebec, Canada. It covers approximately 580,000 km^2^, from 45° to 52° North with annual temperature and precipitation ranging from −2.6 °C to 7.4 °C and from 770 to 1,600 mm, respectively. The area includes various soil substrates and disturbance histories. At the beginning of the 20^th^ century, logging activities were confined to the southern portion of the province (south of 49° north latitude), whereas they currently take place up to 51° North. Each year since the beginning of the 21^st^ century, approximately 0.7% of the managed forest area has been harvested commercially. Besides forest management activities, fires and spruce budworm (*Choristoneura fumiferana* Clemens) outbreaks have been the main disturbances of forest dynamics over the last century^51^.

We compiled tree-ring measurement data acquired by the provincial forest inventory program in Quebec, Canada^52^. These programs include networks of both temporary and permanent sample plots, providing a comprehensive overview of existing forest resources. The third round of forest inventories took place from 1990 to 2002 and provided data from approximately 101,000 sample plots distributed throughout the provincial forest. The fourth round is currently being completed^53^. Circular plots (400 m^2^, radius = 11.28 m) were selected using a stratified random sampling protocol^53^. Along with stand and soil characterization, tree cores were sampled from 1996 to 2012 in both permanent and temporary sample plots according to strict sampling protocols^53,54^. The sampling procedure at the stand level is not entirely random, and biased toward intensive sampling of large trees. In each temporary sample plot, 3 trees (diameter at breast height [DBH] ≥91 mm) were sampled at a height of 1 m above ground level: 1 tree was selected randomly, another was selected randomly among the 4 biggest trees (in DBH) of the dominant species, and the last had the diameter closest to the mean diameter of the dominant tree species. In each permanent sample plot, up to 9 trees were sampled: 5 trees were selected randomly, 2 were selected randomly among the 4 biggest trees (in DBH) of the dominant species, 1 had the diameter closest to the mean diameter of the dominant tree species, and the last had a basal area at breast height closest to the 30^th^ percentile of the distribution of stem basal area for the dominant species. In all cases, only one core per tree was sampled. In 2012, the database included tree-ring measurements of close to 365,000 trees from 42 species and 121,000 plots. Complete core sampling and measurements, mainly conducted to estimate site index, were limited to coniferous species and to the most abundant shade-intolerant deciduous species that generally form even-aged stands (*Betula papyrifera* Marshall *and Populus sp*.). Cores were dried, glued to a wooden tray, and sanded according to standard procedure^55^. Ring boundaries were first detected and identified under binocular magnification, then measured to the nearest 0.001 mm with the WinDendro image analysis system for tree-ring measurement (Regent Instruments Inc.). From this database, we first focused on black spruce (*Picea mariana*), the most abundant boreal tree species, which represents approximately one quarter of the 363,933 core samples (Fig. 1), then checked how patterns held up for the other 12 most abundant boreal species. To avoid problems inherent to stem asymmetry and bark thickness variability over time, we filtered samples using individual cumulative growth instead of field diameter measurements, in order to select only trees cumulating more than 45.5 mm of growth (sampling DBH threshold of 91 mm).

## Supplementary information

Supplementary Information

## Data Availability

Raw provincially-owned data are available from the “Direction des inventaires forestiers du Ministère des Forêts, de la Faune et des Parcs” upon request. The Direction may be contacted at: services.clientele@mffp.gouv.qc.ca.
