# Supplementary material for: Large apparent growth increases in boreal forests inferred from tree-rings are an artefact of sampling biases
Source: Sci Rep. 2019 May 2;9:6832. doi: 10.1038/s41598-019-43243-1 (PMC6497877; doi:10.1038/s41598-019-43243-1)
Supplement: Supplementary file 1 — Supplementary Information [file 41598_2019_43243_MOESM1_ESM.pdf]

Supplemental Information for Large apparent growth increases in boreal forests inferred from tree-rings are an artefact of sampling biases

Louis Duchesne [a,1]

Daniel Houle [a,b]

Rock Ouimet [a]

Liam Caldwell [c]

Manuel Gloor [c]

Roel Brienen [c]

[a] Ministère des Forêts, de la Faune et des Parcs, Direction de la recherche forestière, 2700 Einstein Street, Quebec City, Quebec, G1P 3W8, Canada.

[b] Consortium on Regional Climatology and Adaptation to Climate Change (Ouranos), 550 Sherbrooke Street West, Montreal, Quebec, H3A 1B9, Canada.

[c] School of Geography, University of Leeds, Leeds LS2 9JT, UK.

[1] Corresponding author. Tel. (418) 643-7994 ext. 6537; e-mail: louis.duchesne@mffp.gouv.qc.ca

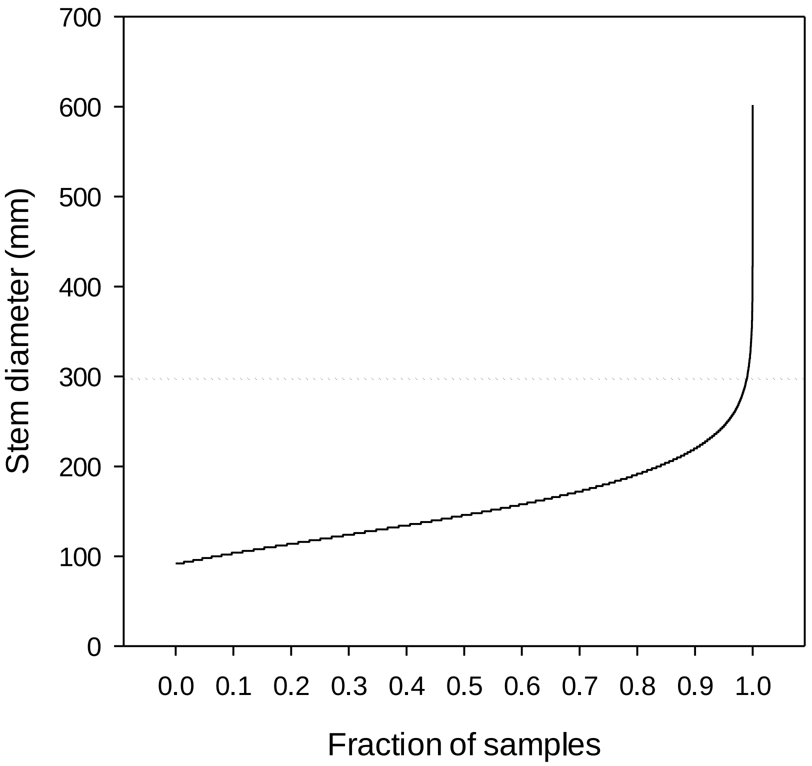

Figure S1. Quantile plot of the stem diameter of sampled black spruce trees with stem diameter at breast height > 90 mm, n = 85,982). The horizontal dotted line (diameter = 298 mm) corresponds to the 99th percentile of the distribution.

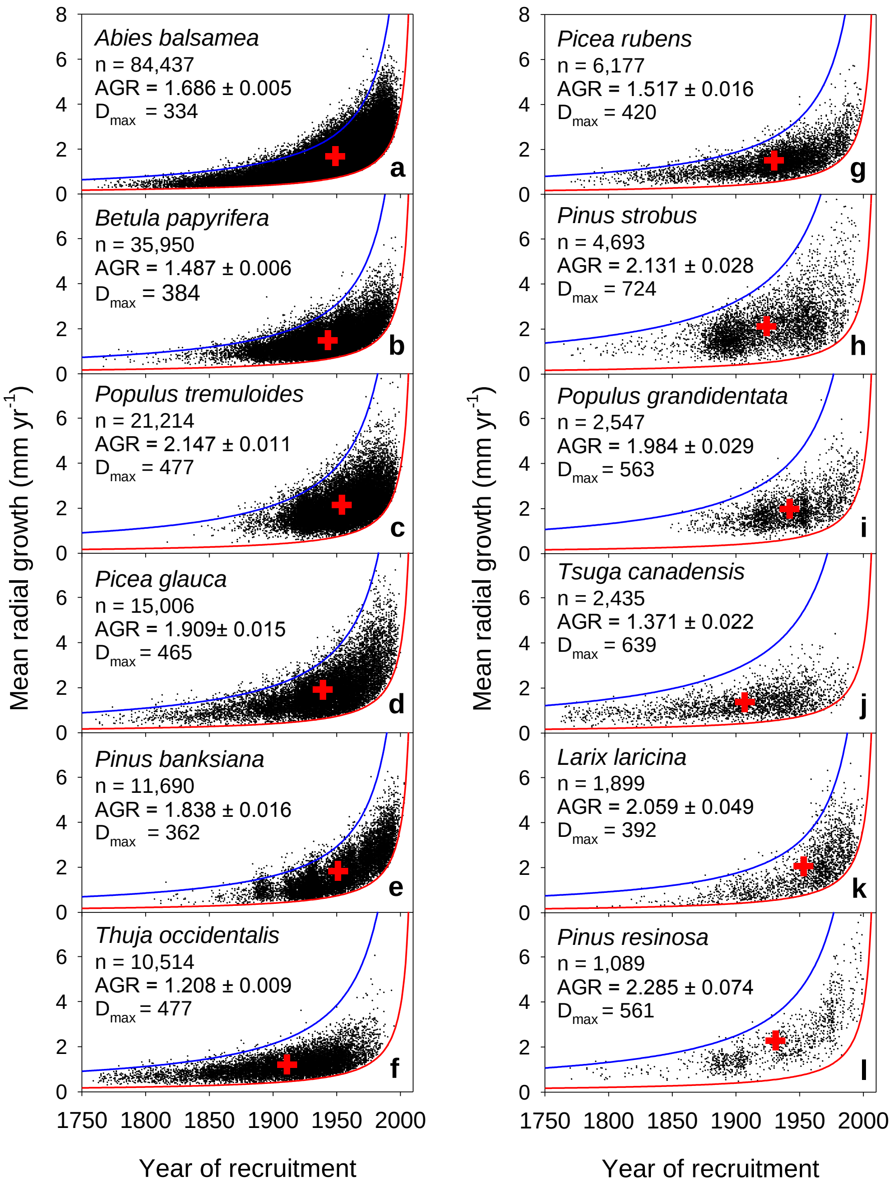

Figure S2. Lifetime mean annual radial growth of individual trees grouped by species and aligned by year of recruitment (pith year). Sample size (n), mean annual growth rate (AGR, mm yr^{-1}) and its 95% confidence interval, and maximum diameter ($D_{max,}$ mm) are given for each species. See the legends of Fig. 3 and Fig. 4 for definition of red cross, blue line and red line. Older tree cohorts are biased toward a higher proportion of slow-growing trees, while younger generations are biased toward a higher proportion of fast-growing trees. These biases induce spurious trends in historical growth rate reconstructions.

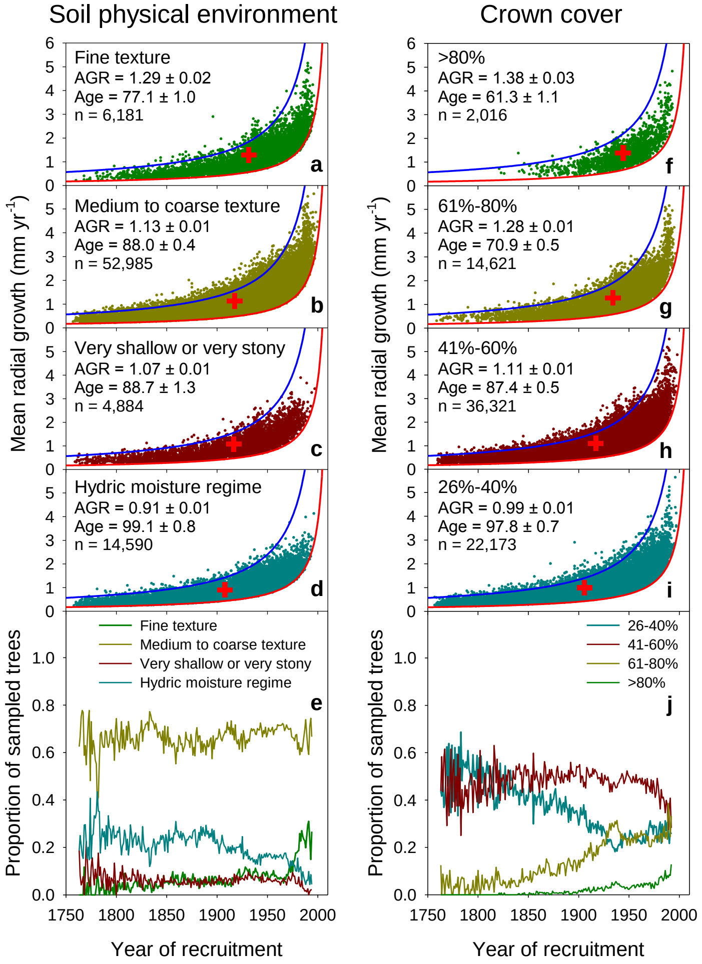

Figure S3. Lifetime mean annual radial growth of individual black spruce trees aligned by year of recruitment (pith year, panels a–d, f–i) and proportion of sampled trees from yearly generations (panels e, j) according to soil physical environment (left) and percentage of crown cover (right). Red crosses indicate the mean annual radial growth rates and mean year of recruitment for each subset. Mean annual growth rate (AGR, mm yr^{-1}), mean tree age (yr) and their 95% confidence intervals, and sample size (n) are given for each subset. See the legend of Fig. 3 for definition of blue and red lines. Older tree cohorts are biased toward a higher proportion of slow-growing trees originating from less productive sites, while younger generations are biased toward a higher proportion of fast-growing trees from more productive sites. These biases induce spurious trends in historical growth rate reconstructions. The proportion series were truncated when sample replication dropped below 25.

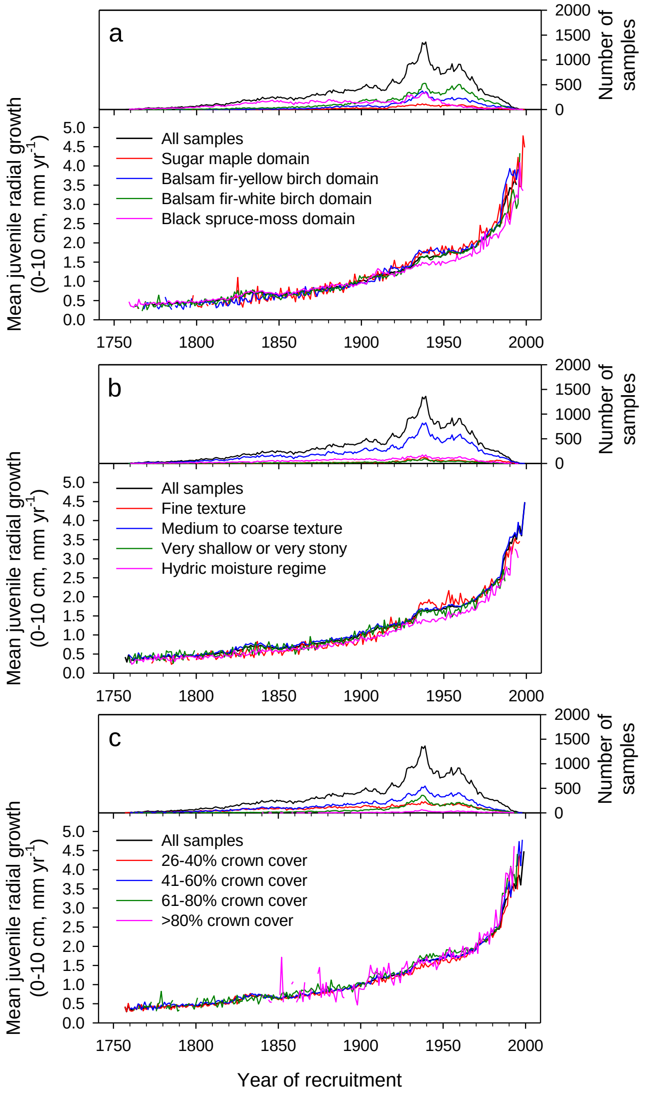

Figure S4. Time series of average mean annual radial growth of trees at the juvenile stage (0–10 cm diameter) from yearly successive generations of black spruce trees stratified by bioclimatic domain (a), soil physical environment (b), and stand density (c). Upper graphs illustrate the number of samples in each generation. Analysis shows that growth rates and recruitment years exhibit high variability within each bioclimatic domain, soil physical environment, and stand cover class. For each of these subsets, this results in biases of similar magnitude to the bias in the full tree-ring dataset. Thus the bias results entirely from the differences in survival rates between fast and slow growers within a tree population (which exists even within sites). It cannot be avoided by limiting the analysis to a smaller number of sites of similar growth conditions.

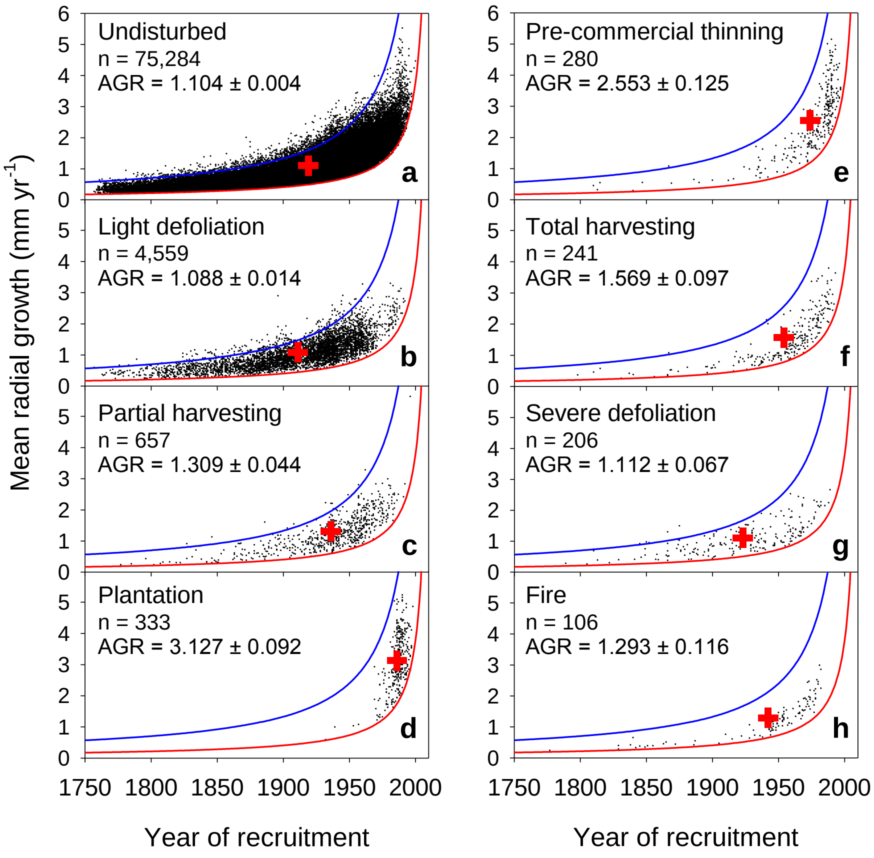

Figure S5. Lifetime mean annual radial growth of individual trees grouped by stand-origin disturbance and intervention (plantation, total harvesting, severe insect defoliation, and fire) or partial disturbance eliminating from 25 to 75% of the stand basal area (light insect defoliation, partial harvesting, and pre-commercial thinning), and aligned by year of recruitment (pith year). Samples size (n), and mean annual growth rate (AGR, mm yr^{-1}) and its 95% confidence intervals, are given for each species. See the legends of Fig. 3 and Fig. 4 for definitions of red cross, blue line and red line. Older tree cohorts are biased toward a higher proportion of slow-growing trees, while younger generations are biased toward a higher proportion of fast-growing trees. Analysis revealed that trees from managed, disturbed and undisturbed stands all exhibit biases of similar magnitude.

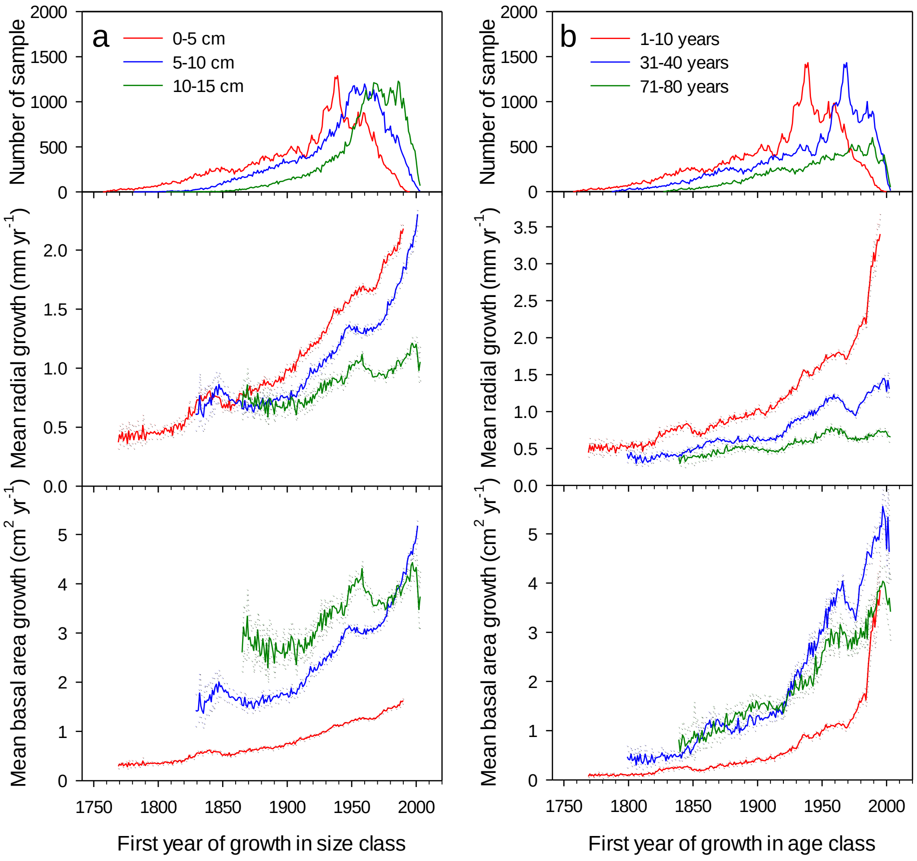

Figure S6. Time series of average mean annual radial and basal area growth of trees in distinct stem diameter (a) and age (b) classes. Data were aligned by the first year of growth in that particular class. Upper panels show sample size. Dotted lines delimit 95% confidence intervals. Only growth data from trees with a minimum of 10 years within these size or age classes were considered. Growth series were truncated when sample replication dropped below 25.

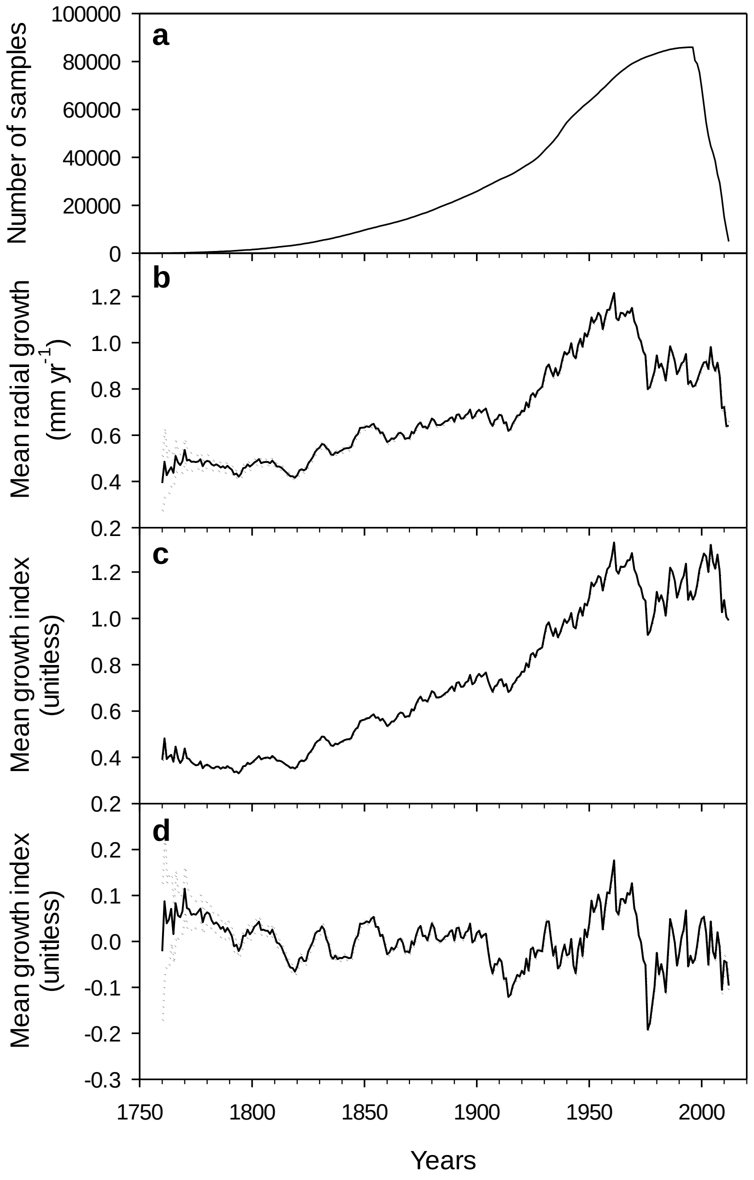

Figure S7. Number of samples (a), time series of mean annual radial growth (b), mean radial growth index following regional curve standardization[35] (c), mean radial growth index following linear regression standardization (d) of black spruce trees. Dotted lines delimit the ±95% confidence intervals. The upper graph illustrates the number of samples available over the years. Annual radial growth and growth indices were averaged according to the year of their formation. The increasing growth trend for b and c is of smaller amplitude than the reported trend

associated with yearly cohorts (Fig. 2), as ring width (or indices) of older, slow-growing trees would be part of the entire chronology and averaged with the young, fast-growing trees that progressively entered the sample forming the chronology. The mean chronologies of annual radial growth and growth indices derived from regional curve standardization indicate that radial growth rate doubled from the 1760s to the 1980s. In contrast, such increasing trend is not perceptible in mean chronologies computed from residuals of independently modelled individuals (panel d) because most of inter-tree growth differences have been discarded.
